# Review on the Risk Assessment of Heavy Metals in Malaysian Clams

**DOI:** 10.1155/2015/905497

**Published:** 2015-04-28

**Authors:** Md. Faruk Hossen, Sinin Hamdan, Md. Rezaur Rahman

**Affiliations:** Faculty of Engineering, Universiti Malaysia Sarawak, 94300 Kota Samarahan, Sarawak, Malaysia

## Abstract

The current review discusses the levels of six heavy metals in different clam species from 34 sites of Malaysian coasts. The concentrations (*µ*g/g dry weight) of these heavy metals ranged around 0.18–8.51, 0.13–17.20, 2.17–7.80, 0.84–36.00, 24.13–368.00, and 177.82–1912.00 for Cd, Pb, Ni, Cu, Zn, and Fe, respectively. It was observed that the concentrations of metals slightly depend on different clam species but mostly depend on site locations. According to Malaysian Food Regulation (1985), about 30% and more than 50% sites are safe from Cd and Pb contamination, respectively, and also the clam species from the other populations studied were safe for consumption.

## 1. Introduction

Heavy metals pollution has been a hot issue in marine environmental studies for many years. Even though metals occur naturally in the environment, due to the anthropogenic inputs which originate from various human activities the concentrations have been rising [1–3]. Increased coastal population, rapid urbanization, oil and gas production, tourism development, heavy rainfall throughout the year, and various economic activities have created numerous environmental and ecological problems in Malaysia's coastal areas, including beach erosion, resource depletion and environmental degradation, and destruction of natural habitats [4–7]. Bivalve mollusks such as clams, oyster, and cockles are found on the mangrove mudflats and intertidal sandy beaches and are well-known accumulator of heavy metals and have been widely used as bioindicator for monitoring heavy metal pollution in aquatic environment in Malaysian coasts [3, 5, 8–13].

According to the Department of Fisheries Malaysia (2005), in 2005-2006, the Malaysia Fisheries Directory documented that there are approximately 27 species of bivalves in Malaysian coastal areas. Clams like other marine bivalves are benthic filter feeders; they are a potential biomonitor of the bioavailability of toxic metal contamination in the estuary [14]. These animals ingest metal-enriched particles directly, thereby giving an indication of the bioaccumulation ability of metals [15, 16]. The increases in anthropogenic activities contribute to the accumulation of hazardous chemicals, such as heavy metals, in the environment [3, 9, 17]. Heavy metal discharged into the environment rapidly associates with particulates and ultimately settles in bottom sediments of water bodies, either direct discharge or surface run-offs [18]. Most of the living organisms need small amount of essential metals such as Fe, Mn, Cu, and Zn for essential processes such as growth [12, 19]. However, all these metals will give harmful effects when exceeding the standard limits [20]. The nonessential metals such as Cd, Pb, Ni, and Cr are toxic even at relatively low concentration and not essential for metabolic activities [12, 21]. The abundance of heavy metal may jeopardize human health due to the consumption of contaminated bivalves [3]. For examples, Cd may cause human carcinogen; Pb can damage blood circulation and excessive intake of Zn may cause electrolyte imbalance and lethargy [22–24].

The main objective in this review is to study the concentration of heavy metal in clam species from different coasts of Malaysia. In this study, heavy metals (Cd, Pb, Ni, Cu, Zn, and Fe) in 12 clam species from 34 sites of Malaysian coasts have been considered and an assessment of human health risks has been conducted comparing those with the food criteria set by different countries.

## 2. Discussion

**Figure 1:**
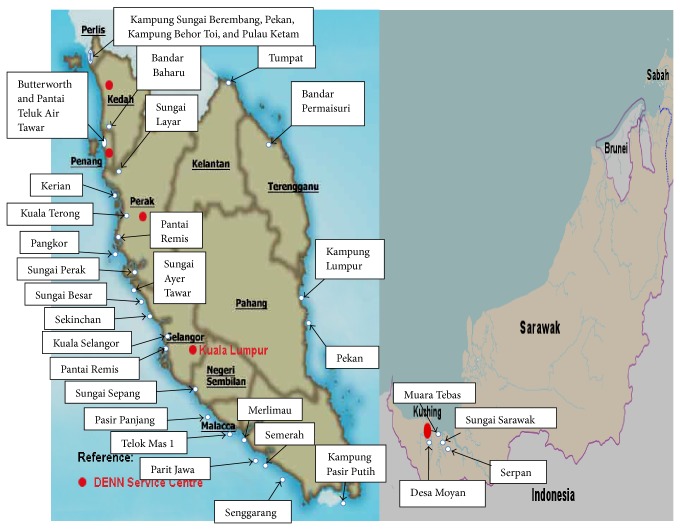
Map of Malaysian coasts showing sampling sites.

The heavy metals such as Cd, Pb, As, and Hg are known as nonessential metals. They are toxic even in small amount and harmful to human health. Harmful metals released by the human activities will be accumulated in marine organisms through the food web. Hence, human health risks might be caused by consumption of seafood contaminated by toxic metals. Normally, human body requires some of heavy metals such as Cu, Zn, Fe, and Ni although sometimes Ni acts as toxic metal. These metals are known as essential metals. However, the essential metals can be toxic if taken in large quantities [20, 25, 26]. In this study, heavy metals such as Cd, Pb, Ni, Cu, Zn, and Fe in total soft tissues of 12 clam species from 34 sites (Figure 1) of Malaysian coasts have been discussed.

### 2.1. Cadmium

It was observed that the range of Cd concentration was 0.18–8.51 *μ*g/g (dry weight) in different clam species from different coasts of Malaysia (Table 1). The highest value was observed in* Scapharca broughtonii* from Pantai Remis (Perak), while the lowest value was observed in* Anadara granosa* from Tumpat (Kelantan). The Cd levels in clam species from most of the sites exceeded the maximum permissible limits set by Malaysian Food Regulation (1985) and ICES (1988) but the species from Bandar Baharu (Kedah), Merlimau (Malacca), Semerah (Johor), Tumpat (Kelantan), Pekan and Tanjung Lumpur (Pahang), Bandar Permaisuri (Terengganu), and Desa Moyan and Serpan (Sarawak) exhibited lower values than safety limits. Only three species from Kampung Sungai Berembang and Pulau Ketam, Perlis, and Pantai Remis, Perak, exceeded the maximum permissible limit set by Brazilian Ministry of Health (ABIA, 1991) but were lower than the permissible limits for human consumption set by Food and Drug Administration of the United States (USFDA, 1990) and the Australian Legal Requirements for Food Safety (NHMRC, 1987) (Table 2). However, it was observed that Cd in clam species from six sites is within the lowest safety limits (1.01–2.00 *μ*g/g) set by Malaysian Food Regulation (1985), International Council for the Exploration of the Seas (ICES, 1988), and Hong Kong Environmental Protection Department (HKEPD, 1997) while Cd in clams from 17 sites is within the 2nd highest safety limits (2.01–5.00 *μ*g/g) set by Brazilian Ministry of Health (ABIA, 1991). On the other hand, only three species from Kampung Sungai Berembang (Perlis), Pulau Ketam (Perlis), and Pantai Remis (Perak) are within the highest safety limits (5.01–10.00 *μ*g/g) set by the Australian Legal Requirements for Food Safety (NHMRC, 1987).

### 2.2. Lead

The concentrations of Pb range from 0.13 to 19.10 *μ*g/g (dry weight) (Table 1). The highest value was observed in* Scapharca broughtonii* from Pantai Remis (Perak), while the lowest value was observed in* Anadara granosa* from Pekan (Pahang). The Pb levels in clams from 14 sites exceeded the maximum permissible limits set by Malaysian Food Regulation (1985) and International Council for the Exploration of the Seas (ICES, 1988) while species from 9 sites exceeded the maximum permissible limits set by Brazilian Ministry of Health (ABIA, 1991) and Ministry of Public Health, Thailand (MPHT, 1986). But six clam species from Pantai Remis (Perak), Pasir Panjang (Negeri Sembilan), Telok Mas 1 (Malacca), Parit Jawa and Kampung Pasir Putih (Johor), and Muara Tebas (Sarawak) exceeded all of the maximum permissible limits (Table 2). However, it was observed that Pb levels in clams from more than 50% sites are below the safety limits (Table 1) but in Sungai Sarawak (Sarawak) and Sekinchan and Kuala Selangor (Selangor) are within the lowest safety limits (2.01–6.00 *μ*g/g) set by Malaysian Food Regulation (1985), International Council for the Exploration of the Seas (ICES, 1988), and Hong Kong Environmental Protection Department (HKEPD, 1997) and only in three species from Pantai Remis (Selangor), Sungai Sepang (Selangor), and Pantai Remis (Perak) are within the highest safety limits (6.01–11.50 *μ*g/g) set by Ministry of Public Health, Thailand (MPHT, 1986), Food and Drug Administration of the United States (USFDA, 1990), and Brazilian Ministry of Health (ABIA, 1991) while that from 6 sites mentioned earlier exceeded all of those safety limits (Table 2).

### 2.3. Nickel

Nickel normally occurs at very low concentrations in the environment. It was observed that the range of Ni was 1.25–7.80 *μ*g/g (dry weight) in different clam species from different coasts of Malaysia (Table 1). The highest value was observed in* Polymesoda erosa* from Parit Jawa, Johor, and the lowest value was observed in* Pholas orientalis* from Sekinchan, Selangor. Ni in clams from Sungai Sepang (Selangor), Pantai Remis (Selangor), Kampung Pasir Putih (Johor), Parit Jawa (Johor), and Pantai Remis (Perak) were higher than 5.00 *μ*g/g.

### 2.4. Copper

Copper is an essential element for human health. The range of Cu concentration was 0.84–36.00 *μ*g/g (dry weight) in different clam species from different coasts of Malaysia (Table 1). The highest value was observed in* Polymesoda expansa* from Kampung Pasir (Johor) and the lowest value was observed in the same species from Sungai Sarawak (Sarawak). The Cu levels from all of the sites were below the maximum permissible limits set by Malaysian Food Regulation (1985), Ministry of Public Health, Thailand (MPHT, 1986), Australian Legal Requirements for Food Safety (NHMRC, 1987), International Council for the Exploration of the Seas (ICES, 1988), Food and Drug Administration of the United States (USFDA, 1990), and Brazilian Ministry of Health (ABIA, 1991) while Cu in the same ones from Kampung Pasir Putih (Johor) exceeded the maximum permissible limits set by Malaysian Food Regulation (1985) (Table 2).

### 2.5. Zinc

Zinc is also an essential element like copper. It was observed that the Zn concentrations in different clam species from different coasts of Malaysia ranged widely from 24.13 to 368.00 *μ*g/g (dry weight) (Table 1). The highest value was observed in* Polymesoda expansa* from Kampung Pasir Putih (Johor), while the lowest value was observed in* Meretrix meretrix* from Sungai Sarawak (Sarawak). The Pb levels of clam species from almost 10 sites exceeded the maximum permissible limits set by Malaysian Food Regulation (1985) and only* Polymesoda erosa* and* Polymesoda expansa* from Sungai Sepang, Selangor, and Kampung Pasir, Johor, respectively, exceeded the second highest maximum permissible limits set by Brazilian Ministry of Health (ABIA, 1991) but Zn levels from all of those sites were below the highest maximum permissible limits set by Ministry of Public Health, Thailand (MPHT, 1986), and Australian Legal Requirements for Food Safety (NHMRC, 1987) (Table 2).

However, Zn value in clams from 6 sites was within the lowest safety limits (100.01–250.00 *μ*g/g) set by Malaysian Food Regulation (1985) and Brazilian Ministry of Health (ABIA, 1991) while Zn in clams only from Kampung Pasir (Johor) and Sungai Sepang (Selangor) was above this lowest safety limit but below the highest safety limits (667.01–750.00 *μ*g/g).

### 2.6. Iron

Iron is an essential nutrient metal required for human. It was observed that the Fe concentration ranged widely from 177.82 to 1912.00 *μ*g/g (dry weight) in different clam species from different coasts of Malaysia (Table 1). The highest value was observed in* Polymesoda erosa* from Telok Mas 1 (Malacca), while the lowest value was observed in* Solen regularis* from Sungai Sarawak (Sarawak). However, very high values of Fe were observed in clams from Telok Mas 1 (Malacca), Kampung Pasir (Johor), Parit Jawa (Johor), and Sungai Sepang (Selangor) which were 1912, 1454, 1307, and 1111, *μ*g/g respectively.

## 3. Health Risk Assessment

In this study, an assessment on human health risks has been conducted by comparing the levels of the heavy metals found in the total soft tissues of clam species (Table 1) with the food criteria set by different countries (Table 2).

Cadmium is one of the environmental contaminants which can promote serious damage to human health. It was observed that Cd levels within the lowest safety limits (1.01–2.00 *μ*g/g) may be due to the fact that most of those samples sites were within the vicinity of agricultural areas of mostly large oil palm plantations heavy in pesticides and herbicides used [2, 5] while the levels are within the 2nd highest safety limits (2.01–5.00 *μ*g/g). On the other hand, Cd levels within the highest safety limits (5.01–10.00 *μ*g/g) may be due to the influence of external discrete sources like industrial activities, agriculture runoff, and other anthropogenic inputs [3, 39]. From this study, it is revealed that Cd may cause possible toxicological risks and heavy metal related diseases, such as Parkinson's and Wilson's diseases [40] due to long term consumption especially for the population of Kampung Sungai Berembang (Perlis), Pulau Ketam (Perlis), and Pantai Remis (Perak).

Lead is also one of the environmental contaminants. It was observed that Pb levels in clam species from more than 50% sites are below the safety limits (Table 1) but those from Sungai Sarawak (Sarawak) and Sekinchan and Kuala Selangor (Selangor) are within the lowest safety limits (2.01–6.00 *μ*g/g) which indicated that these sites are slightly polluted. Only three species from Pantai Remis (Selangor), Sungai Sepang (Selangor), and Pantai Remis (Perak) are within the highest safety limits (6.01–11.50 *μ*g/g) set while Pb levels in clams from 6 sites mentioned earlier exceeded all of those safety limits (Table 2) and this maybe resulted from burning of fossil fuels from boats used for fishing and also leisure activities [1, 6]. This may be the cause of neurological deficits such as mental retardation in children and kidney disease such as interstitial nephritis to adults and also contribute to hypertension and cardiovascular disease [41] to the consumers in these coastal areas after long term consumption.

Nickel normally occurs at very low concentrations in the environment and it can cause variety of pulmonary adverse health effects [42]. The levels of Ni in clams from Sungai Sepang (Selangor), Pantai Remis (Selangor), Kampung Pasir (Johor), Parit Jawa (Johor), and Pantai Remis (Perak) were higher than 5.00 *μ*g/g (Table 1). The cause may be that human activities such as metal mining, smelting, refining, fossil fuel combustion, and solid waste disposal are the significant sources of this metal discharge to the environment and large amount may be transferred to marine environment through municipal sewage effluent containing industrial waste [6, 15, 43]. Ni has no definite safety limits but high values may lead to serious health problems, including respiratory system cancer, and it can also cause a skin disorder known as nickel-eczema [44].

Copper is an essential nutrient and is necessary for the synthesis of hemoglobin [45] and its deficiency can result in blood and nervous system disorders [46]. It was observed that Cu in clams from all sites was below the safety limits (Table 1) indicating that it should not pose an acute toxicological risk to the consumers. But Cu only in* Polymesoda expansa* from Kampung Pasir, Johor, exceeded the maximum permissible limits set by Malaysian Food Regulation (1985) (Table 2). This higher value may come from the paddy field activities where pesticides are used to prevent the insects' attack [3, 6] and can cause liver and kidney disease [47] and it may lead to stunted human growth due to long term consumption [48].

Zinc is also an important metal in human nutrition and fulfills many biochemical functions in human metabolism. Zn deficiency in human organism leads to several disorders, but an excessive Zn intake can cause acute adverse effects [49]. However, Zn value in calms from 6 sites is within the lowest safety limits (100.01–250.00 *μ*g/g) indicating that these sites may be close to boating activities, fish landing, restaurants, and sightseeing view place [3], while Zn in clams only from Kampung Pasir (Johor) and Sungai Sepang (Selangor) is above the lowest safety limit but below the highest safety limits (667.01–750.00 *μ*g/g). This may be from antifouling paint and incidental discharges of fuel, oil from boats, ship, and also municipal sewage [50], because Zn has been used as an anticorrosion agent and its ability to get speedy oxidation might enhance the level of zinc in these two sites. This will lead to a tendency in the organism to accumulate the high amount of Zn in its soft tissue [11]. In addition these sites are all in the vicinity of ports busy with navigational activities and cargo handling particularly petroleum and petroleum products [5]. Thus, high levels of zinc result in decreased cytochrome oxidase activity in the heart and liver as well as catalase in the liver [51] due to long term consumption.

Iron is an essential nutrient metal required for human but its deficiency is frequently associated with anemia [27, 28]. Smelting and refining of metals, steel manufacturing, and metal plating that mobilized iron by human activities may lead to the Fe contamination in marine environment [43]. However, very high values of Fe were observed in clams from Telok Mas 1 (Malacca), Kampung Pasir (Johor), Parit Jawa (Johor), and Sungai Sepang (Selangor) which were 1912, 1454, 1307, and 1111 *μ*g/g, respectively [6]. It has no definite safety limits like other metals but very high values due to the tendency of Fe to bind with organic matter in marine environment [29, 30] and also from those human activities.

In this study, it was found that the levels of metal slightly depend on species (Table 1). It was evidenced that different organisms display a range of capacities for the accumulation of metals, varying from accumulators to accumulators of certain elements [6, 13, 31–36]. Although the bioavailability of contaminants in the environment is complicated issue which involves many chemical, physical, and biological factors [37, 38], the use of the soft tissues of different clam species might provide a better insight into the bioavailability of metals. On the other hand, it was observed that the levels of metal mostly depend on site locations (Table 1). However, according to Malaysian Food Regulation (1985), about 30% sites are safe from Cd contamination while more than 50% sites are safe from Pb contamination.

## 4. Conclusion

In the present study, it was found that the clam species from different sites accumulated heavy metals at different concentrations. It was also observed that the concentrations of metals slightly depend on different clam species but mostly depend on site locations. The results revealed that the clams from mentioned sites have higher values than the food safety limits which should be avoided in order to avoid any possible toxicological risks and heavy metal related diseases, such as Parkinson' disease, Wilson's disease, and Hallervorden-Spatz disease, due to long term consumption. On the other hand, according to Malaysian Food Regulation (1985), about 30% and more than 50% sites are safe from Cd and Pb contamination, respectively, and also the clam species from the other populations studied were safe for consumption.

## Figures and Tables

**Table 1:** Mean concentration of metals (*μ*g/g dry weight) in total soft tissue of different clam species from Malaysia.

Locations	Clam species	Cd	Pb	Ni	Cu	Zn	Fe	References
Perlis								
Kampung Sungai Berembang	*Glauconome virens *	—	—	1.25	8.76	74.62	572.60	[15]
Pekan	*Marcia marmorata *	3.40	—	—	22.80	88.70	—	[3]
Kampung Behor Toi	*Marcia marmorata *	1.90	—	—	14.90	77.50	—	
Kampung Sungai Berembang	*Marcia marmorata *	7.10	—	—	23.90	89.70	—	
Pulau Ketam	*Marcia marmorata *	5.20	—	—	24.70	116.50	—	
Kedah								
Bandar Baharu	*Anadara granosa *	0.67	0.16	—	2.01	57.20	—	[5]
Sungai Layar	*Glauconome virens *	—	—	2.19	11.05	119.28	706.60	[15]
Penang								
Butterworth	*Anadara granosa *	3.43	1.08	—	3.27	98.79	—	[5]
Pantai Teluk Air Tawar	*Glauconome virens *	—	—	2.05	11.02	132.06	—	[15]
Perak								
Kerian	*Anadara granosa *	2.60	0.92	—	1.67	56.43	—	[5]
Kuala Terong	*Anadara granosa *	2.49	0.93	—	3.18	77.21	—	
Pangkor	*Anadara granosa *	2.14	0.79	—	2.42	73.41	—	
Sungai Perak	*Anadara granosa *	1.49	1.33	—	2.57	69.82	—	
Pantai Remis	*Scapharca broughtonii *	8.51	19.10	5.26	4.54	67.80	782.00	[6]
Pantai Remis	*Trisidos kiyonoi *	2.64	8.00	4.93	5.84	61.50	501.00	
Selangor								
Sungai Ayer Tawar	*Anadara granosa *	2.67	1.78	—	3.07	136.03	—	[5]
Sungai Besar	*Anadara granosa *	4.43	1.61	—	2.17	158.00	—	
Kuala Selangor	*Anadara granosa *	2.66	3.04	—	2.94	99.70	—	
Sekinchan	*Pholas orientalis *	2.31	3.96	2.17	16.86	40.43	291.15	[10]
Pantai Remis	*Pholas orientalis *	1.75	8.21	6.17	22.55	37.76	292.75	
Sungai Sepang	*Polymesoda erosa *	2.96	6.80	5.07	15.70	343.00	1111.00	[6]
Negeri Sembilan								
Pasir Panjang	*Donax faba *	3.96	12.60	3.65	7.23	51.20	654.00	[6]
Malacca								
Merlimau	*Anadara granosa *	0.62	0.91	—	9.10	98.72	—	[5]
Telok Mas 1	*Polymesoda erosa *	4.23	11.60	4.29	5.91	249.00	1912.00	[6]
Johor								
Semerah	*Anadara granosa *	0.82	1.43	—	1.96	53.22	—	[5]
Senggarang	*Anadara granosa *	1.45	0.60	—	2.67	65.91	—	
Parit Jawa	*Polymesoda erosa *	3.34	12.20	7.80	6.50	222.00	1307.00	[6]
Kampung Pasir Putih	*Polymesoda expansa *	3.59	17.20	6.04	36.00	368.00	1454.00	
Kelantan								
Tumpat	*Anadara granosa *	0.18	0.99	—	1.84	41.80	—	[5]
Pahang								
Pekan	*Anadara granosa *	0.65	0.13	—	4.92	104.02	—	[5]
Tanjung Lumpur	*Solen brevis *	0.67	1.61	—	8.64	87.74	415.20	[11]
Terengganu								
Bandar Permaisuri	*Anadara granosa *	0.35	0.45	—	2.19	64.50	—	[5]
Sarawak								
Muara Tebas	*Solen *spp.	1.64	13.47	—	4.11	82.82	522.07	[1]
Desa Moyan	*Solen regularis *	0.85	—	—	6.75	—	263.50	[2]
Serpan	*Solen regularis *	0.88	—	—	8.05	—	601.25	
Sungai Sarawak	*Polymesoda expansa *	1.15	2.89	—	0.84	62.24	295.31	[13]
Sungai Sarawak	*Meretrix meretrix *	2.15	2.23	—	1.57	24.13	250.73	
Sungai Sarawak	*Solen regularis *	2.35	4.85	—	2.21	27.08	177.82	

**Table 2:** Guidelines on heavy metals (*μ*g/g dry weight) for food safety set by different countries [27–38].

Countries	Cd	Cu	Pb	Zn	Ni	Fe
Malaysian Food Regulation (1985)	1.00	30.0	2.00	100.00	—	—
International Council for the Exploration of the Seas (ICES, 1988)	1.80	—	3.00	—	—	—
Brazilian Ministry of Health (ABIA, 1991)	5.00	150	10.0	250.00	—	—
Ministry of Public Health, Thailand (MPHT, 1986)	—	133	6.67	667.00	—	—
Food and Drug Administration of the United States (USFDA, 1990)	25.0	—	11.50	—	—	—
Australian Legal Requirements for Food Safety (NHMRC, 1987)	10.0	350	—	750.00	—	—
Hong Kong Environmental Protection Department (HKEPD, 1997)	2.00	—	6.00	—	—	—
